# Virological Changes of Chronic Hepatitis B Patients with Minimally Elevated Levels of Alanine Aminotransferase: A Meta-Analysis and Systematic Review

**DOI:** 10.1155/2022/7499492

**Published:** 2022-11-16

**Authors:** Xiyao Chen, Xingrong Zheng, Hewei Wu, Boxiang Zhang, Liang Peng, Chan Xie

**Affiliations:** ^1^Department of Infectious Diseases, The Third Affiliated Hospital of Sun Yat-sen University, 600# Tianhe Road, Guangzhou 510630, Guangdong, China; ^2^Guangdong Provincial Key Laboratory of Liver Disease, Guangzhou, China

## Abstract

**Background:**

Chronic hepatitis B (CHB) patients with normal or minimally increased levels of alanine aminotransferase (ALT) are still at the risk of hepatocellular carcinoma, cirrhotic events, and mortality. However, there is a debate over the initiation of antiviral treatment for these patients. This systematic review and mate-analysis aimed to explore this problem.

**Methods:**

MEDLINE (PubMed), EMBASE, Cochrane Central Register of Controlled Trials, and Web of Science databases were systematically searched for retrieving relevant studies with risk ratios (RRs) or risk differences (RDs) for virological changes between antivirus-treated and no antivirus-treated CHB patients with ALT levels less than two-fold of the upper limit of normal. Retrieved data ranged from January 1990 to October 2020.

**Results:**

Of 6783 abstracts screened, 9 studies met the criteria for inclusion in the systematic review and had a low risk of bias. Among studies that were involved in the meta-analyses, it was found that the rates of HBsAg loss (RR = 12.22, 95% confidence interval (CI): 4.28–34.95, *P* < 0.001), HBsAg seroconversion (RR = 19.90, 95% CI: 2.75–144.09, *P*=0.003), and undetectable HBV DNA (RR = 11.89, 95% CI: 2.44–57.89, *P*=0.002) were both higher in the antiviral treatment group compared with placebo or no treatment group. Subgroup analysis suggested that patients who received interferon (IFN)-based therapy were more inclined to achieve HBsAg loss (*P*=0.010), HBsAg seroconversion (*P*=0.020), and HBeAg loss (*P*=0.002).

**Conclusion:**

From a sizable population, it was revealed that CHB patients with normal or minimally increased levels of ALT could benefit from the antiviral therapy, especially those who received IFN-based treatment.

## 1. Introduction

Chronic hepatitis B (CHB) virus infection remains a worldwide health burden, afflicting approximately 257 million people [1]. Up to 40% of untreated CHB patients progress to cirrhosis, and these patients are at the risk of developing decompensated cirrhosis and hepatocellular carcinoma (HCC) [2, 3]. Studies have shown that a significant proportion (40%) of CHB patients have normal or minimally increased levels of alanine aminotransferase (ALT), while they may still be at the risk of HCC, cirrhotic events, and mortality in patients with CHB-related cirrhosis [4–7]. Hence, timely treatment can delay disease progression and improve the prognosis.

At present, it is still an area of ongoing controversy on whether CHB patients with mildly raised ALT levels (more than one-fold, while less than two-fold of the upper limit of normal (ULN)) should be treated. The American Association for the Study of Liver Diseases (AASLD, updated in 2018) [8] and the Asian-Pacific guideline (updated in 2015) [9] concluded that the threshold of the ALT level for initiating antiviral therapy is no less than two-fold of the ULN. Conversely, the European Association for the Study of the Liver (EASL, updated in 2017) recommended treatment for patients with a serum ALT concentration of more than the ULN and hepatitis B virus (HBV) DNA >2000 IU/L [1]. In the current Chinese guideline, patients with the ALT level more than the ULN and detectable HBV DNA should receive treatment [10]. Therefore, the agreement on the initiation of antiviral therapy was not reached worldwide. In a retrospective cohort study with involvement of 3624 untreated CHB patients that was conducted in South Korea, it was revealed that patients whose conditions did not meet therapeutic indications for EASL, AASLD, and other authoritative guidelines still had a cumulative 5-year HCC incidence of 2.1–3.2% [11]. At present, the notion that whether this group of CHB patients should be treated remains controversial and whether they can benefit from the antivirus therapy is still undiscovered.

Hepatitis B surface antigen (HBsAg) loss with or without HBsAg seroconversion is regarded as a functional cure and the ultimate endpoint for CHB therapy [1, 8, 9]. For patients who achieved partial cure, HBV DNA is undetectable in their serum [12]. The present study aimed to identify the proportion of HBsAg loss with or without the seroconversion to hepatitis B surface antibody (HBsAb) in CHB patients with only normal or minimally elevated ALT levels. The secondary objective was to identify the proportions of undetectable HBV DNA, hepatitis B e antigen (HBeAg) loss, and HBeAg seroconversion.

## 2. Results

### 2.1. Search Results

**Figure 1:**
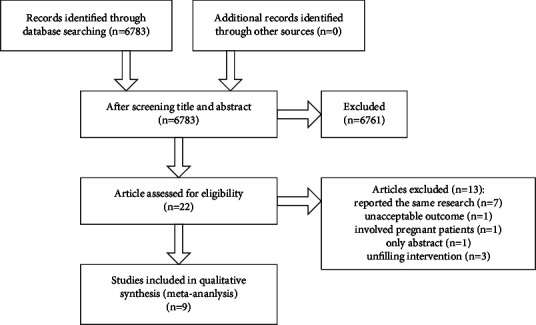
Flowchart showing selection of articles for meta-analysis.

Totally, 6783 articles were retrieved through searching in the four databases mentioned below, of which 512 articles were eliminated because of duplicate publication. After screening of titles and abstracts, the full texts of 22 studies were downloaded to assess their eligibility. Finally, 7 articles [13–20] and 2 abstracts [21] which met the eligibility criteria are included in this meta-analysis (Figure 1). The details of the study selection are shown in Table 1. During the analysis, one of the abstracts was published and our collected data were renewed subsequently [18].

### 2.2. Study and Patients' Characteristics

In the primary analysis, there were a total of 898 patients in the 9 included studies. Most of the included studies were randomized controlled trials (RCTs), except for 2 studies [13, 15], which did not assign patients randomly. In addition to one study published in 2002 [20], the year of publication of the other studies was between 2014 and 2021. Of note, patients were treated exclusively with nucleotide analogs (tenofovir) in Hsu et al. [18], nucleoside analogs (entecavir) in Tseng et al. [17], nucleotide analogs plus nucleoside analogs (tenofovir and emtricitabine) in Chan et al. [16], and IFN combined with NAs in 5 studies. In 2 studies [19, 20], two or more different regimens were used in the experimental group, whereas only one study [20] reported the outcome of each regimen. In 3 studies [13, 14, 21], patients in the experimental group were given IFN at the beginning, and in 2 studies [13, 14], they additionally received NAs based on their conditions.

It was found that 2 studies [17, 18] enrolled both HBeAg-positive and HBeAg-negative patients, regardless of their HBeAg status, in which enrolled subjects were grouped in one research [17]. However, the remaining 7 studies involved either in HBeAg-negative or HBeAg-positive patients. As for 4 studies that enrolled HBeAg-positive patients, one study [20] reported HBV DNA level in pg/mL, and the others [14–16] reported in IU/mL and enrolled patients with high viral load (>20000 IU/mL). Furthermore, regarding 3 studies [13, 19, 21] that enrolled only HBeAg-negative individuals, the level of HBV DNA ≤20000 IU/ml (all reported in IU/mL unit) was covered in the inclusion criterion.

Most of the studies mainly enrolled male adult patients. Nevertheless, 2 HBV-infected children with immune-tolerant characteristic and HBV postpartum women were enrolled, respectively. The majority of patients were Asian except for those in 3 studies [16, 19, 20]. Besides, 2 trials measured the outcomes at 192 weeks [16] and 144 weeks [18], and 7 studies performed measurement within 96 weeks.

### 2.3. Results of Meta-Analysis

Of the 9 studies, 6, 5, 4, 5, and 6 studies reported the outcomes of HBsAg loss, HBsAg seroconversion, HBeAg loss, HBeAg seroconversion, and undetectable HBV DNA, respectively. Stratified by the treatment strategy, treatment duration, or baseline parameters, subgroup analysis was performed to assess the association between antiviral therapy and each endpoint in the meta-analysis using a random-effects model.

### 2.4. HBsAg Loss

**Figure 2:**
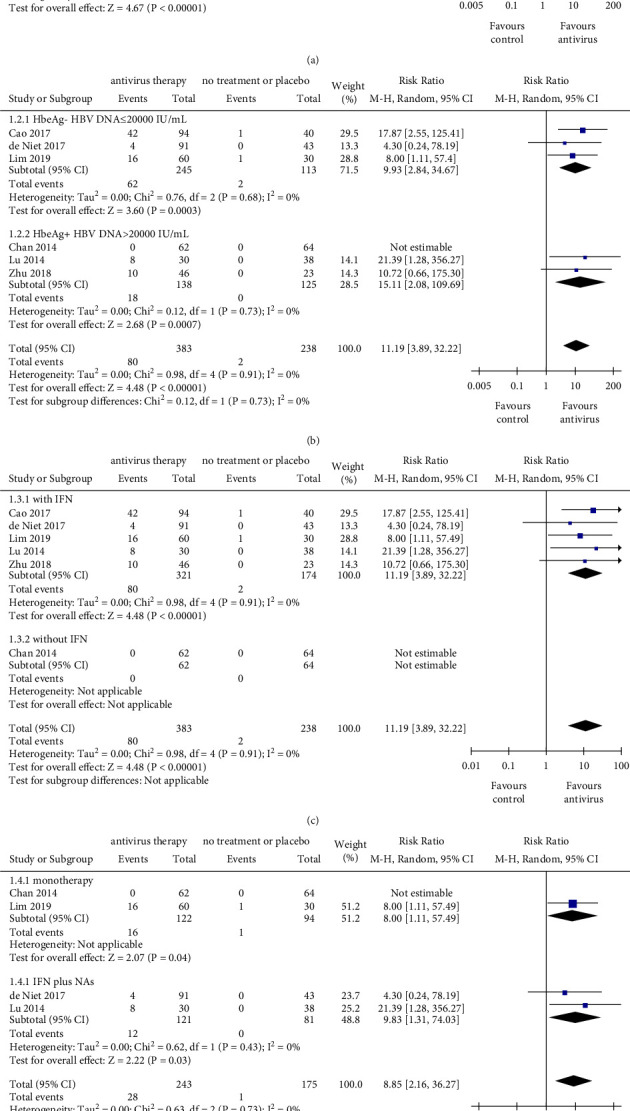
The outcomes of HBsAg loss. (a) Pooled risk ratio for HBsAg loss between the antiviral therapy group and control group. (b) Subgroup analysis stratified by patients' baseline parameters (the status of HBeAg and the level of HBV DNA). (c) Subgroup analysis stratified by therapeutic regimen with or without IFN. (d) Subgroup analysis stratified by monotherapy and combined therapy (IFN plus NAs). NAs, nucleos(t)ide analogs; IFN, interferon; CI, confidence interval; monotherapy exclusively included NAs or IFN. The size of square represents the weight of each study, and the vertical dotted line represents the pooled rate.

Compared with the control group, antiviral therapy was associated with a significantly higher incidence of HBsAg loss (RR = 12.22, 95% CI: 4.28–34.95, *P* < 0.001) (Figure 2(a)). Then, it was attempted to explore how the characteristics of viral replication could affect HBsAg loss, and patients were stratified by the status of HBeAg and HBV DNA. Both subjects with HBeAg-positive and HBV DNA >20000 IU/mL (RR = 15.11, 95% CI: 2.08–109.69, *P*=0.007) and subjects with HBeAg-negative and HBV DNA ≤20000 IU/mL (RR = 9.93, 95% CI: 2.84–34.67, *P* < 0.001) exhibited a superior effect of HBsAg loss than that of the control one. The difference between the two subgroups (subjects with HBeAg-positive and HBV DNA>20000 IU/mL subgroup and subjects with HBeAg-negative and HBV DNA ≤20000 IU/mL subgroup) was also not significant (*P*=0.730) (Figure 2(b)). Besides, in studies with IFN treatment, antiviral therapy was associated with a significantly higher incidence of HBsAg loss (RR = 11.19, 95% CI: 3.89–32.22, *P* < 0.001). In addition, there was no event of HBsAg loss in the interferon-free studies, regardless of antiviral or viral event (Figure 2(c)). When the outcomes were calculated as RD, there was a remarkable difference in HBsAg loss between IFN-based subgroup and IFN-free subgroup (*P*=0.010) (Supplementary Figure 1). These results indicated that IFN therapy had a higher incidence of HBsAg loss in CHB patients. Furthermore, stratified by treatment strategy, both combination therapy (RR = 9.83, 95% CI: 1.31–74.03, *P*=0.030) and monotherapy (RR = 8.00, 95% CI: 1.11–57.49, *P*=0.040) exhibited a superior effect of HBsAg loss than that of the control one. However, the difference between the subgroups (monotherapy subgroup and combination therapy subgroup) was not significant (*P*=0.890) (Figure 2(d)). In conclusion, patients with antiviral treatment were inclined to have the clearance of HBsAg.

### 2.5. HBsAg Seroconversion

**Figure 3:**
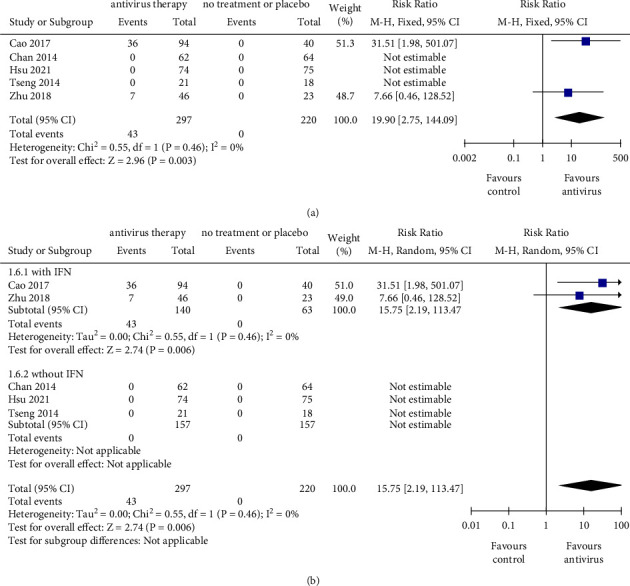
The outcomes of HBsAg seroconversion. (a) Pooled risk ratio for HBsAg seroconversion between the antiviral therapy group and control group. (b) Subgroup analysis stratified by therapeutic regimen with or without IFN. IFN, interferon; CI, confidence interval. The size of square represents the weight of each study, and the vertical dotted line represents the pooled rate.

Compared with nonantiviral therapy, antiviral therapy was associated with a significantly higher incidence of HBsAg seroconversion (RR = 19.90, 95% CI: 2.75–144.09, *P*=0.003) (Figure 3(a)). Among 5 studies that reported the events of HBsAg seroconversion, all events happened in the combination group. In studies with IFN-based treatment, antiviral therapy was associated with a significantly higher incidence of HBsAg seroconversion (RR = 15.75, 95% CI: 2.19–113.47, *P*=0.006). In contrast, in studies that adopted IFN-free regimen, no event of HBsAg seroconversion occurred (Figure 3(b)). When the outcomes were calculated as RD, there was a significant difference between IFN-based subgroup and IFN-free subgroup (*P*=0.020) (Supplementary Figure 2). Collectively, patients with antiviral therapy, especially those undergoing IFN treatment, were more inclined to have HBsAg seroconversion.

### 2.6. HBeAg Loss

**Figure 4:**
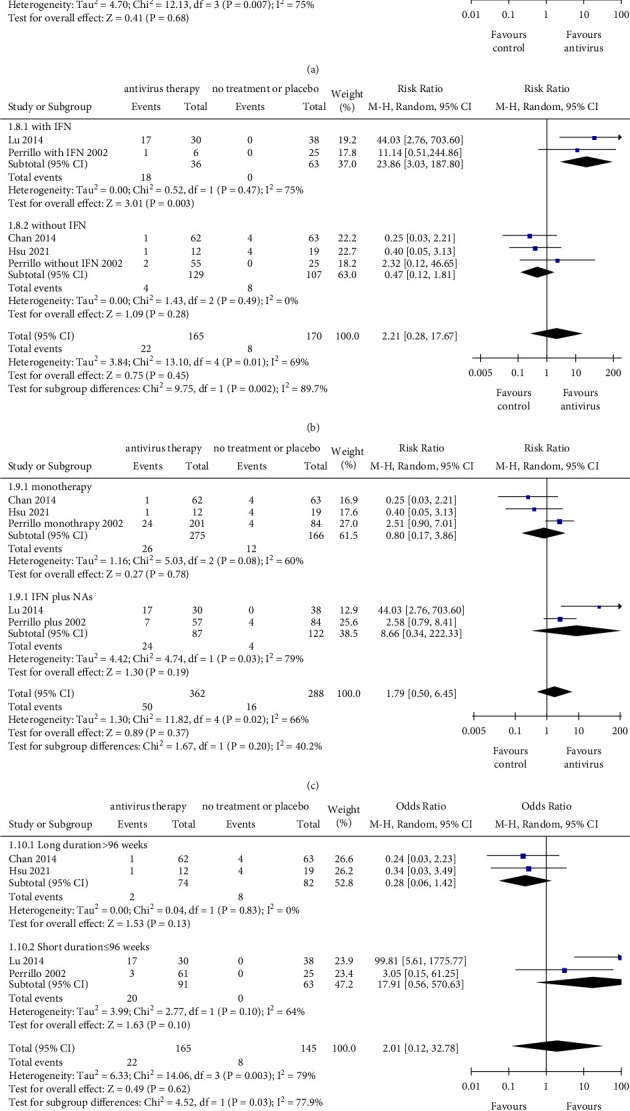
The outcomes of HBeAg loss. (a) Pooled risk ratio for HBeAg loss between the antiviral therapy group and control group. (b) Subgroup analysis stratified by therapeutic regimen with or without IFN. (c) Subgroup analysis stratified by monotherapy and combined therapy (IFN plus NAs). (d) Subgroup analysis stratified by therapy duration with threshold of 96 weeks. NAs, nucleos(t)ide analogs; IFN, interferon; CI, confidence interval; monotherapy group exclusively included NAs or IFN. The size of square represents the weight of each study, and the vertical dotted line represents the pooled rate.

Compared with nonantiviral treatment, antiviral therapy was associated with no significant difference in the rate of HBeAg loss (RR = 1.68, 95% CI: 0.14–19.67, *P*=0.680; with a noticeable heterogeneity *I*^2^ = 75%) (Figure 4(a)). Therefore, sensitivity analysis was performed to find the source of heterogeneity. The results showed that with the removal of Lu's study (2014), the heterogeneity could reduce to a degree where it was not significant (Supplementary Figure 3). In studies that involved IFN-based treatment, antiviral therapy was associated with a significantly higher incidence of HBeAg loss (RR = 23.86, 95% CI: 3.03–187.80, *P*=0.003). In contrast, in studies that adopted IFN-free regimen, there were no significant difference between the two groups in HBeAg loss (RR = 0.47, 95% CI: 0.12–1.81, *P*=0.280). Besides, the difference between the subgroups (IFN-based subgroup and IFN-free subgroup) was statistically significant (*P*=0.002) (Figure 4(b)). Both combination therapy (RR = 8.66, 95% CI: 0.34–222.33, *P*=0.190; with a noticeable heterogeneity *I*^2^ = 79%) and monotherapy (RR = 0.80, 95% CI: 0.17–3.86, *P*=0.780; with a noticeable heterogeneity *I*^2^ = 60%) exhibited the same effect of HBsAg loss than that of the control one. In addition, the difference between the two subgroups (monotherapy subgroup and combination therapy subgroup) was not significant (*P*=0.200) (Figure 4(c)). To further investigate the association between therapy duration and HBeAg loss, subgroup analysis was conducted in short-term (≤96 weeks) and long-term (>96 weeks). Although a significant difference was found between the two subgroups (*P*=0.030), neither the long duration subgroup nor the other subgroup showed a significant difference between the antiviral therapy group and control group (Figure 4(d)). Collectively, only patients who underwent IFN-based antiviral therapy were more inclined to have HBeAg loss, and patients may not benefit from a long-term treatment.

### 2.7. HBeAg Seroconversion

**Figure 5:**
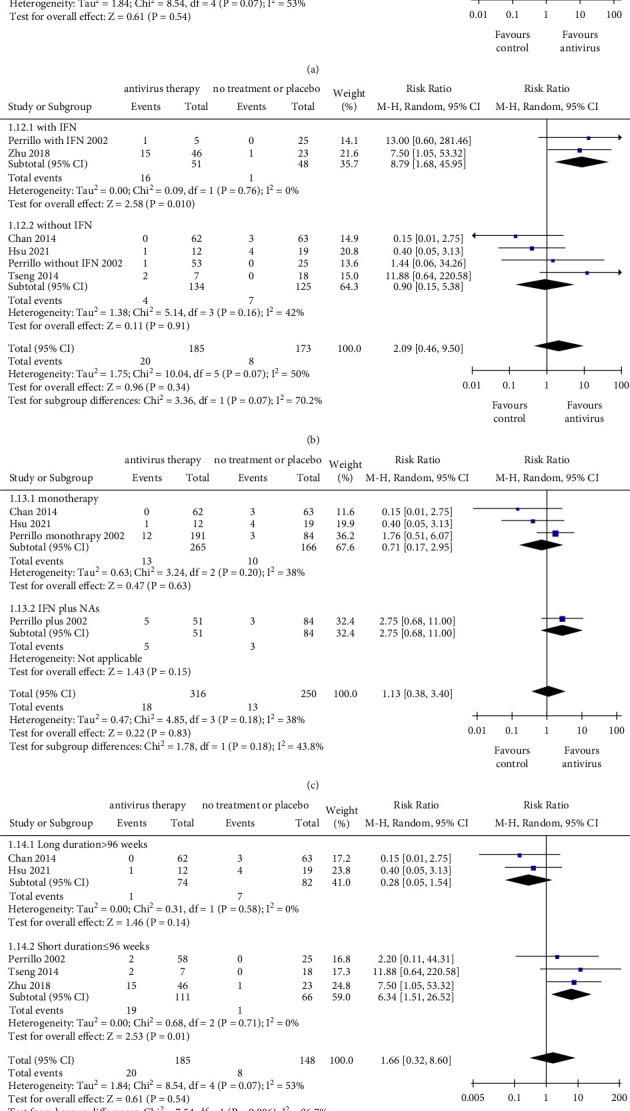
The outcomes of HBeAg seroconversion. (a) Pooled risk ratio for HBeAg seroconversion between the antiviral therapy group and control group. (b) Subgroup analysis stratified by therapeutic regimen with or without IFN. (c) Subgroup analysis stratified by monotherapy and combined therapy (IFN plus NAs). (d) Subgroup analysis stratified by therapy duration with threshold of 96 weeks. NAs, nucleos(t)ide analogs; IFN, interferon; CI, confidence interval; monotherapy group exclusively included NAs or IFN. The size of square represents the weight of each study, and the vertical dotted line represents the pooled rate.

Compared with nonantiviral treatment, antiviral therapy was associated with no significant difference in the rate of HBeAg seroconversion (RR = 1.66, 95% CI: 0.32–8.60, *P*=0.540; with a noticeable heterogeneity *I*^2^ = 53%) (Figure 5(a)). In studies that adopted IFN-based treatment, antiviral therapy was associated with a significantly higher incidence of HBeAg seroconversion (RR = 8.79, 95% CI: 1.68–45.95, *P*=0.010). In contrast, in studies that adopted interferon-free regimen, there was no significant difference in HBeAg seroconversion between the two groups (RR = 0.90, 95% CI: 0.15–5.38, *P*=0.910). Subgroup analysis showed no significant difference between IFN-based subgroup and IFN-free subgroup (*P*=0.070) (Figure 5(b)). Both combination therapy (RR = 2.75, 95% CI: 0.68–11.00, *P*=0.150) and monotherapy (RR = 0.71, 95% CI: 0.17–2.95, *P*=0.630) exhibited the same effect of HBsAg loss compared with the control one. Moreover, the difference between the two subgroups (monotherapy subgroup and combination treatment subgroup) was not significant (*P*=0.180) (Figure 5(c)). In addition, stratified by treatment duration, subgroup analysis showed that there was a significant difference between the two subgroups (long-term subgroup and short-term subgroup) (*P*=0.006). Only in the short-term subgroup, antiviral therapy exhibited a superior effect of HBeAg seroconversion than that of the control one (RR = 6.34, 95% CI: 1.51–26.52, *P*=0.010) (Figure 5(d)). Generally, only patients who underwent IFN-based antiviral therapy were more inclined to have HBeAg seroconversion. Besides, the extension of the treatment duration did not promote the occurrence of HBeAg seroconversion.

### 2.8. Undetectable HBV DNA

**Figure 6:**
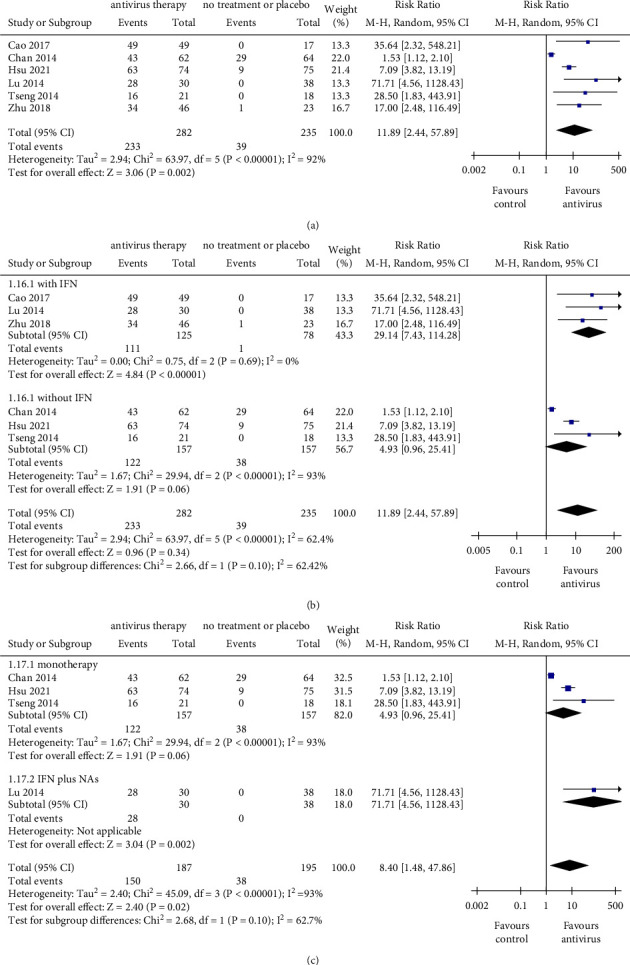
The outcomes of undetectable HBV DNA. (a) Pooled risk ratio for undetectable HBV DNA between the antiviral therapy group and control group. (b) Subgroup analysis stratified by therapeutic regimen with or without IFN. (c) Subgroup analysis stratified by monotherapy and combined therapy (IFN plus NAs). NAs, nucleos(t)ide analogs; IFN, interferon; CI, confidence interval; monotherapy group exclusively included NAs or IFN. The size of square represents the weight of each study, and the vertical dotted line represents the pooled rate.

Compared with nonantiviral treatment, antiviral therapy was associated with a significantly higher incidence of undetectable HBV DNA (RR = 11.89, 95% CI: 2.44–57.89, *P*=0.002; with a noticeable heterogeneity *I*^2^ = 92%) (Figure 6(a)). Sensitivity analysis with removing Chan's study (2014) for undetectable HBV DNA revealed consistent results with the primary meta-analysis, while heterogeneity was reduced to a degree where it was not significant (Supplementary Figure 3). In studies that adopted IFN-based treatment, antiviral therapy was associated with a significantly higher incidence of undetectable HBV DNA (RR = 29.14, 95% CI: 7.43–114.28, *P* < 0.001); whereas in studies that adopted IFN-free regimen, there was no significant difference between the two groups in undetectable HBV DNA (RR = 4.93, 95% CI: 0.96–25.41, *P*=0.06). No significant difference was found between the two subgroups (IFN-based subgroup and IFN-free subgroup) (*P*=0.10) (Figure 6(b)). In order to explore whether there was a synergy between IFN and NAs on undetectable HBV DNA, subgroup analysis of monotherapy and combined therapy was conducted. Only in the combined treatment subgroup, antiviral therapy exhibited a superior effect than that of the control one (RR = 71.71, 95% CI: 4.56–1128.43, *P*=0.002). However, the difference between the two subgroups (monotherapy subgroup and combination treatment subgroup) was insignificant (*P*=0.100) (Figure 6(c)). Collectively, patients who underwent antiviral treatment were inclined to have undetectable HBV DNA.

## 3. Discussion

In this systematic review, we evaluated the proportion of HBsAg loss with or without the seroconversion to HBsAb in CHB patients with only normal or minimally elevated ALT levels. It was revealed that antiviral therapy increased the rate of achieving HBsAg loss, HBsAg seroconversion, and undetectable HBV DNA compared with no or placebo treatment. IFN exhibited a more important role in HBsAg loss with or without HBsAg seroconversion than NAs. Besides, no synergism was found between IFN and NAs in virological response.

There was a consensus that antiviral therapy should be actively administered in patients with elevated ALT levels (>2 ULN), cirrhosis, and liver cancer, while there was a controversy over antiviral therapy in CHB patients with normal or mildly elevated ALT levels. Studies have suggested that CHB patients with normal or low ALT levels have a certain histological damage, and they may eventually develop liver failure, cirrhosis, or HCC [22, 23]. A retrospective cohort study found that long-term antiviral therapy reduced the incidence of liver cancer in patients with CHB, with no significant association with ALT levels [24]. In addition, a meta-analysis indicated that the rate of fibrosis is more than 40% in patients with CHB and minimal increased ALT levels [25]. Combined with the results of our study, it is likely to conclude that given the possibility of liver disease progression and the available benefits of antiviral therapy, the threshold for the initiation of treatment must be individualized.

For CHB patients with normal or minimally increased ALT levels, the overall treatment goal is to inhibit and even eliminate hepatitis B virus infection, alleviate necrosis and inflammation, and suppress the disease progression. IFN and NAs are two effective antiviral drugs for CHB patients to delay the disease progression and to improve the long-standing prognosis. A meta-analysis included 24 studies and found that combination therapy took an advantage on promoting HBsAg loss [26]. Another research demonstrated that NAs combined with IFN strategy could improve efficacy on HBeAg seroconversion compared to monotherapy with NAs [27]. However, our study found the equivalence of combination therapy and monotherapy in achieving virological response. Considering the high heterogeneity and small sample size, additional RCTs are required to verify the synergy between IFN and NAs on virological response.

NAs can suppress the replication of hepatitis B virus effectively, while IFN has dual functions on viral inhibition and immunomodulation. In clinical practice, for the vast majority of CHB patients, long-term NA therapy is the top choice for them, apart from the few achieved HBsAg clearance or the conversion to HBsAb. IFN, compared with NAs, is inferior in preventing the virus from replicating itself, while it is superior in achieving HBeAg seroconversion and HBsAg loss [28, 29]. The present study revealed that CHB patients with ALT levels <2 ULN can benefit from IFN-based treatment in achieving primary outcomes. Besides, there was a significant difference in HBeAg clearance, and it could be due to immunomodulatory effects of IFN. Previous studies have demonstrated that IFN-based treatment was associated with greater sustained virological and serological responses and a higher chance of HBsAg loss, and the induced functional cure was durable [30, 31]. However, our research indicated that IFN-based therapy was not superior to IFN-free therapy in achieving HBeAg seroconversion and undetectable HBV DNA, which might be attributed to the same reason as mentioned above.

A previous prospective cohort study revealed that the rate of HBeAg seroconversion increased along with the prolonged treatment [32]. In the subgroup analysis stratified by the treatment duration, short-term treatment, compared with long-term treatment, could improve the efficacy of HBeAg loss and HBeAg seroconversion. The possible reason for the difference was that all the studies included in the long-term treatment subgroup adopted IFN-free strategy and tenofovir treatment, while most of the studies in the short-term treatment subgroup adopted IFN-based strategy, and the difference might stem from IFN treatment.

The present study exhibited the following deficiencies. Firstly, several factors might influence patients' response to the therapy, including demographic characteristic, HBV genotype, HBeAg status, HBV DNA levels, and with or without history of undergoing antiviral treatment. However, the limitation of the available studies impeded further subgroup analysis to explore the influences of the abovementioned factors. Secondly, because the unknown approach for measuring ALT and ULN was not mentioned in some articles, and it was infeasible to evaluate the efficacy of antiviral therapy for CHB patients with normal ALT level. Thirdly, high heterogeneity and limited research studies in each subcohort restricted us from exploring optimal antiviral strategies for CHB patients with ALT level <2 ULN. Fourthly, the small quantity of the available studies and certain methodological limitations, which were related to randomization and blinding processes, diminished the quality of our study.

In general, for CHB patients with ALT level <2 ULN, antiviral therapy could significantly improve the HBsAg loss rate. Through the immune modulatory function of IFN and our results, IFN is highly recommended to CHB patients with ALT level <2 ULN for achieving the objective of functional treatment.

## 4. Methods

This study was conducted and reported according to the PRISMA (Preferred Reporting Items for Systematic Reviews and Meta-Analyses) guidelines. The study protocol was registered in the PROSPERO database (Registration No. CRD42020209639).

### 4.1. Data Sources and Searches

On October 3, 2020, the relevant studies were retrieved by searching four English language databases, such as MEDLINE (PubMed), EMBASE, Cochrane Central Register of Controlled Trials, and Web of Science, from January 1990 to October 2020. The following strategy was applied: (((“Hepatitis B”(Mesh)) OR (“Hepatitis B virus”(Mesh))) OR (“Hepatitis B, Chronic”(Mesh))) AND ((alanine aminotransferase (Title/Abstract)) OR (“Alanine Transaminase”[Mesh))). To avoid missing potentially relevant articles, the additional citations of all retrieved articles were also searched manually.

### 4.2. Study Selection

Studies fulfilling the following specific criteria were considered for inclusion in the primary analysis: (i) patients: both infected with hepatitis B virus and with ALT level ≤2 ULN (ULN shall be subjected to each article reported); (ii) treatment strategy: patients were divided into antiviral therapy group (treated with nucleos(t)ide analogs (NAs) or interferon (IFN)) and control group (no treatment or placebo); and (iii) outcomes: including rates of undetectable HBV DNA, HBeAg loss, HBeAg seroconversion, HBsAg loss, or HBsAg seroconversion. We excluded studies where study cohorts included patients with (i) coinfection with hepatitis C virus, hepatitis D, or human immunodeficiency virus; (ii) remarkable alcohol abuse; or (iii) other liver diseases. Then, four of the authors (C. X. Y, Z. X. R, W. H. W, and Z. B. X) reviewed the articles independently. Information about the source or author of the report was not blinded to the investigators. However, when a decision was made on which studies should be included, two observers (C. X. Y and Z. B. X) analyzed in parallel and without interference. In case of the occurrence of any disagreement, they conferred with other two authors (W. H. W and Z. X. R). The final decision was confirmed by four observers and the senior author (X. C). The decision to include these data was not influenced by the results of the recruited study.

### 4.3. Data Extraction and Study Quality

The modified Newcastle-Ottawa Scale was applied to assess the risk of bias in cohort studies by two reviewers (C. X. Y and Z. B. X) separately. The included studies in the meta-analysis were rated on a scale of zero to 9 points. Only studies that scored six or higher points were regarded as good quality. For each study considered for being included, the following data were fetched from them: (i) study characteristics (first author's name, year of publication, geographic locale, study design, sample size, number of patients included in analysis, and quality score); (ii) patients' demographics (age, gender, HBeAg-positive rate, baseline HBV DNA level, and baseline ALT level); (iii) inclusion and exclusion criteria; (iv) treatment details (i.e., antiviral agent, treatment duration, lower limit of detection of HBV DNA, and data collected at timepoints).

### 4.4. Statistical Analysis

Using the Mantel–Haenszel method, the pooled outcome was calculated as risk ratio (RR) or risk difference (RD) with 95% confidence interval (CI) for various indicators reported as dichotomous variables, involving undetectable HBV DNA, HBsAg loss, HBsAg seroconversion, HBeAg loss, and HBeAg seroconversion. The *χ*^2^ test was applied for the exploration of heterogeneity, and statistical significance was set to *P* < 0.05. To quantify heterogeneity, *I*^2^ statistic was taken, with a maximum of 50% that was defined as low heterogeneity, while >50% indicated significant heterogeneity. If there was a high degree of heterogeneity, the random-effects model was used, otherwise, the fix-effects model was utilized. The Review Manager 5.2 software (Cochrane Collaboration, Oxford, UK) was used to calculate RRs and draw the forest plots to display the results of the meta-analysis. The squares around the estimates were proportional to the weights used in the meta-analysis, with horizontal lines that represented 95% CI. Sensitivity analysis was performed by leave-one-out analysis to test the influences of individual studies on aggregate estimates. The following subgroup analyses were carried out, wherever possible: therapeutic strategy (combination therapy (NAs and IFNs), NAs-monotherapy, and IFN-monotherapy; patients who were treated with two or more types of NAs were divided into the monotherapy group), treatment duration, baseline HBeAg status, and baseline HBV DNA level. Once a significant difference was found between the subgroups (test for interaction, *P* < 0.05), the results were reported separately. A formal test was also performed for subgroup interactions.

## Figures and Tables

**Table 1:** Characteristics of included studies and subjects.

First authors	Year	Geographic locale	Study design	Interventions	Sample size	N included in analysis	Sex (male %)	Age (years)	HBeAg positive (%)	Baseline ALT	Baseline HBV DNA	Samples	ALT analysis method	Study timepoints, weeks	NOS score
Perrillo	2002	Multinational	RCT	LAM	55	55	78	Median: 34 (15–73)	100	≤1 × ULN	Median: 98 (pg/mL, LLOD-2,264)	Serum	NR	52	7
				IFN	2	2	81	Median: 32 (16–70)			Median: 111 (pg/mL, LLOD-1,322)				
				LAM + IFN	4	4	71	Median: 34 (15–76)			Median: 94 (pg/mL, LLOD-786)				
				Placebo	25	25	80	Median: 35 (15–67)			Median: 79 (pg/mL, LLOD-1,150)				
Tseng	2014	Taiwan	RCT	ETV	22	22	59	Mean: 45 ± 10	31.8	Mean: 0.6 ± 0.2 ULN	Mean: 5.95 ± 1.3 log_10_ copies/ml	Serum	NR	52	9
				Placebo	20	20	55	Mean: 42 ± 12	42.9	Mean: 0.6 ± 0.2 ULN	Mean: 6.31 ± 1.42 log_10_ copies/ml				
Chan	2014	Multinational	RCT	TDF + placebo	64	64	48.4	Mean: 33 (9.5)	98.4	Mean: 26.9 (14.05) U/L	Mean: 8.42 (0.402) log_10_IU/mL	Serum	NR	192	9
				TDF + FTC	62	62	50	Mean: 33 (11.2)	100	Mean: 26.2 (9.88) U/L	Mean: 8.40 (0.395) log_10_IU/mL				

Lu	2014	China	NRSI	LdT + CPIA	30	30	0	Mean: 28.6	100	≤1 × ULN	>5 × 10^6^ IU/ml	Serum	NR	96	7
				LdT + placebo	38	38	0								

De Niet	2017	Netherlands	RCT	PEG-IFN + ADV	46	46	61	Mean: 44 (12)	0	Median: 27 (21–42) U/L	Mean: 2.65 (1.23) log_10_IU/mL	Serum	NR	72	7
				PEG-IFN + TDF	45	45	47	Mean: 43 (12)		Median: 25 (19–30) U/L	Mean: 2.79 (1.03) log_10_IU/mL				
				No treatment	43	43	60	Mean: 41 (10)		Median: 30 (21–47) U/L	Mean: 2.79 (1.04) log_10_IU/mL				

Cao	2017	China	NRSI	PEG-IFN/+ ADV	94	94	66	38.8 ± 10.0	0	28.3 ± 9.0 IU/L	<2000 IU/mL	Serum	NR	96	7
				No treatment	40	40	65	39.8 ± 10.6		25.4 ± 8.4 IU/L					
Zhu	2018	China	RCT	IFN/+ LAM	46	46	65	Median: 7 (8)	100	Median: 45 (28) U/L	Median: 9.95 × 10^7^ IU/mL	Serum	NR	96	7
				No treatment	23	23	57	Median: 8 (8)	100	Median: 48 (31) U/L	Median: 1.72 × 10^8^ IU/mL				

Lim	2019	Singapore	RCT	PEG-IFN	60	60	NR	21–75	0	≤1 × ULN	≤20000 IU/ml	Serum	NR	48	6
				No treatment	30	30									

Hsu	2021	Taiwan	RCT	TDF	79	74	78	Mean: 45 (39–54)	16	Mean: 53 (45–63) U/L	Mean: 5.26 (4.30–6.23) log_10_IU/mL	Serum	NR	144	9
				Placebo	81	75	80	Mean: 43 (37–51)	26	Mean: 52 (46–66) U/L	Mean: 5.32 (4.41–6.41) log_10_IU/mL				

RCT, randomized controlled trial; NRSI, nonrandomized studies of interventions; LAM, lamivudine; ETV, entecavir; TDF, tenofovir disoproxil fumarate; FTC, emtricitabine; LdT, telbivudine; ADV, adefovir dipivoxil; IFN, interferon; PEG-IFN, pegylated interferon; CPIA, combination of peg IFN and adefovir; ULN, upper limit of normal; NR, not recorded.

## Data Availability

The data used to support the findings of this study are available from the corresponding author upon request.

## Supplementary Materials

Supplementary Materials Supplementary Figure 1: risk difference was used for HBsAg loss under subgroup analysis stratified by therapeutic regimen with or without IFN. CI, confidence intervals. The size of square represents the weight of each study, and the vertical dotted line represents the pooled rate. Supplementary Figure 2: risk difference was used for HBsAg seroconversion under subgroup analysis stratified by therapeutic regimen with or without IFN. CI, confidence intervals. The size of square represents the weight of each study, and the vertical dotted line represents the pooled rate. Supplementary Figure 3: sensitivity analysis. (A) HBeAg loss. (B) HBV DNA undetectable. CI, confidence intervals. The size of square represents the weight of each study, and the vertical dotted line represents the pooled rate.
