# Supplementary figures and images for: Virological Changes of Chronic Hepatitis B Patients with Minimally Elevated Levels of Alanine Aminotransferase: A Meta-Analysis and Systematic Review
Source: Can J Gastroenterol Hepatol. 2022 Nov 16;2022:7499492. doi: 10.1155/2022/7499492 (PMC9683979; doi:10.1155/2022/7499492)

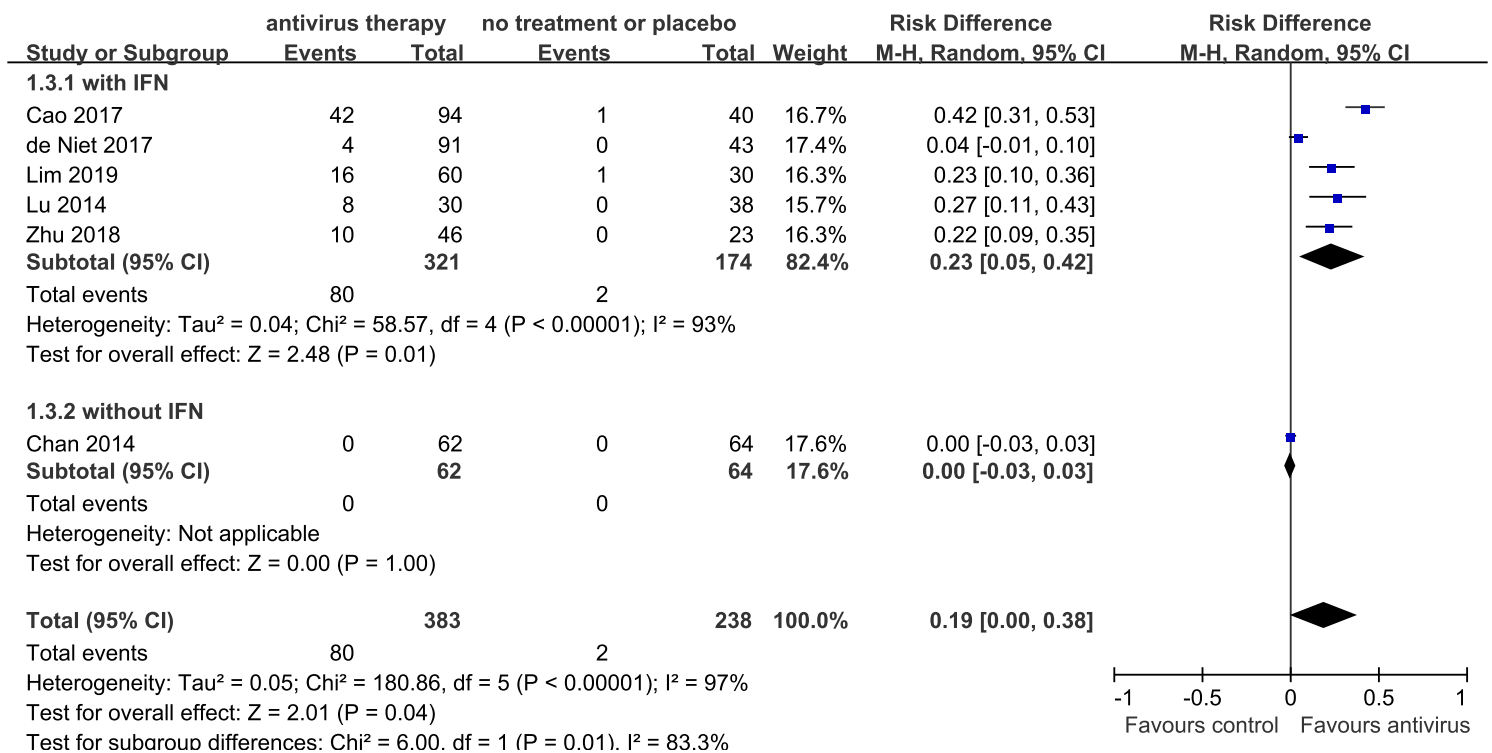

Supplement: Supplementary Materials — Supplementary Figure 1: risk difference was used for HBsAg loss under subgroup analysis stratified by therapeutic regimen with or without IFN. CI, confidence intervals. The size of square represents the weight of each study, and the vertical dotted line represents the pooled rate. Supplementary Figure 2: risk difference was used for HBsAg seroconversion under subgroup analysis stratified by therapeutic regimen with or without IFN. CI, confidence intervals. The size of square represents the weight of each study, and the vertical dotted line represents the pooled rate. Supplementary Figure 3: sensitivity analysis. (A) HBeAg loss. (B) HBV DNA undetectable. CI, confidence intervals. The size of square represents the weight of each study, and the vertical dotted line represents the pooled rate. [file 7499492.f1.zip › Supplementary Figure 1.pdf]

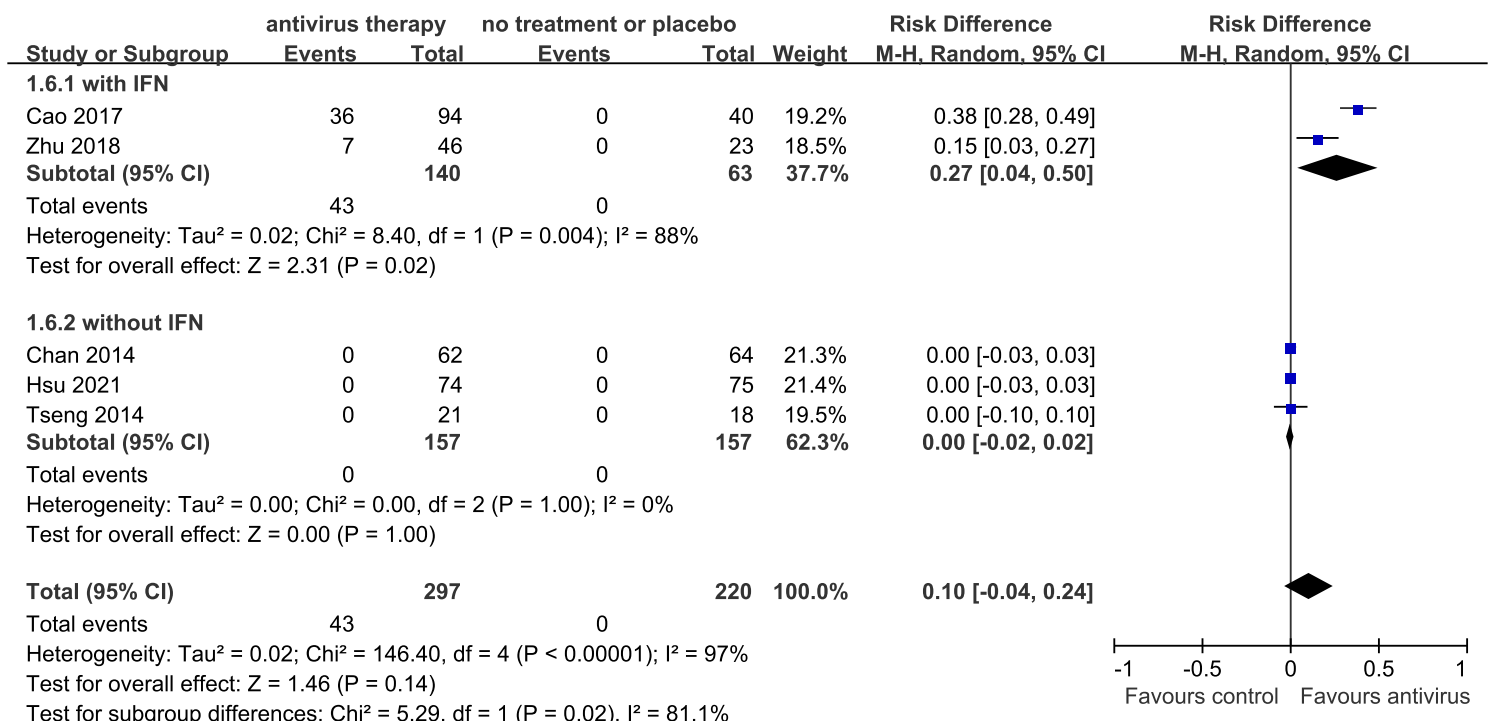

Supplement: Supplementary Materials — Supplementary Figure 1: risk difference was used for HBsAg loss under subgroup analysis stratified by therapeutic regimen with or without IFN. CI, confidence intervals. The size of square represents the weight of each study, and the vertical dotted line represents the pooled rate. Supplementary Figure 2: risk difference was used for HBsAg seroconversion under subgroup analysis stratified by therapeutic regimen with or without IFN. CI, confidence intervals. The size of square represents the weight of each study, and the vertical dotted line represents the pooled rate. Supplementary Figure 3: sensitivity analysis. (A) HBeAg loss. (B) HBV DNA undetectable. CI, confidence intervals. The size of square represents the weight of each study, and the vertical dotted line represents the pooled rate. [file 7499492.f1.zip › Supplementary Figure 2.pdf]

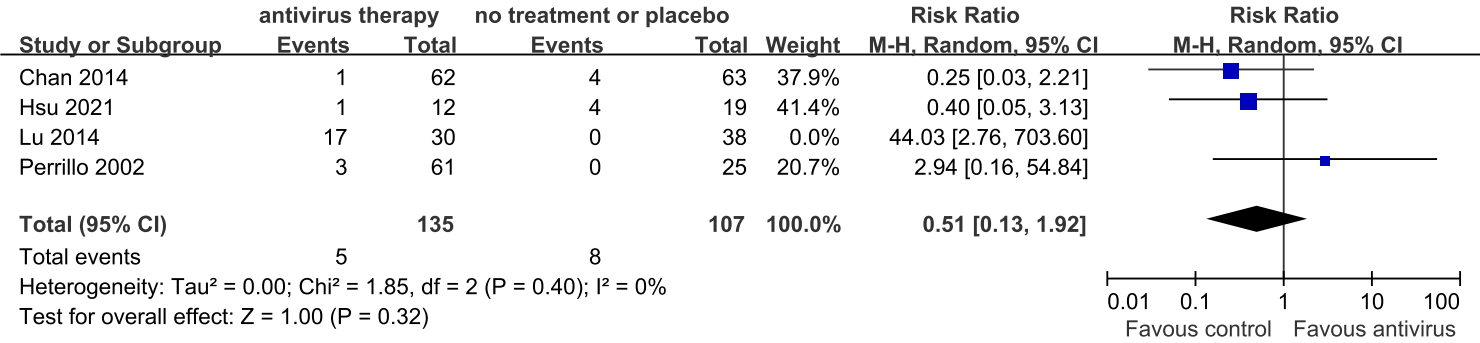

Supplement: Supplementary Materials — Supplementary Figure 1: risk difference was used for HBsAg loss under subgroup analysis stratified by therapeutic regimen with or without IFN. CI, confidence intervals. The size of square represents the weight of each study, and the vertical dotted line represents the pooled rate. Supplementary Figure 2: risk difference was used for HBsAg seroconversion under subgroup analysis stratified by therapeutic regimen with or without IFN. CI, confidence intervals. The size of square represents the weight of each study, and the vertical dotted line represents the pooled rate. Supplementary Figure 3: sensitivity analysis. (A) HBeAg loss. (B) HBV DNA undetectable. CI, confidence intervals. The size of square represents the weight of each study, and the vertical dotted line represents the pooled rate. [file 7499492.f1.zip › Supplementary Figure 3A.pdf]

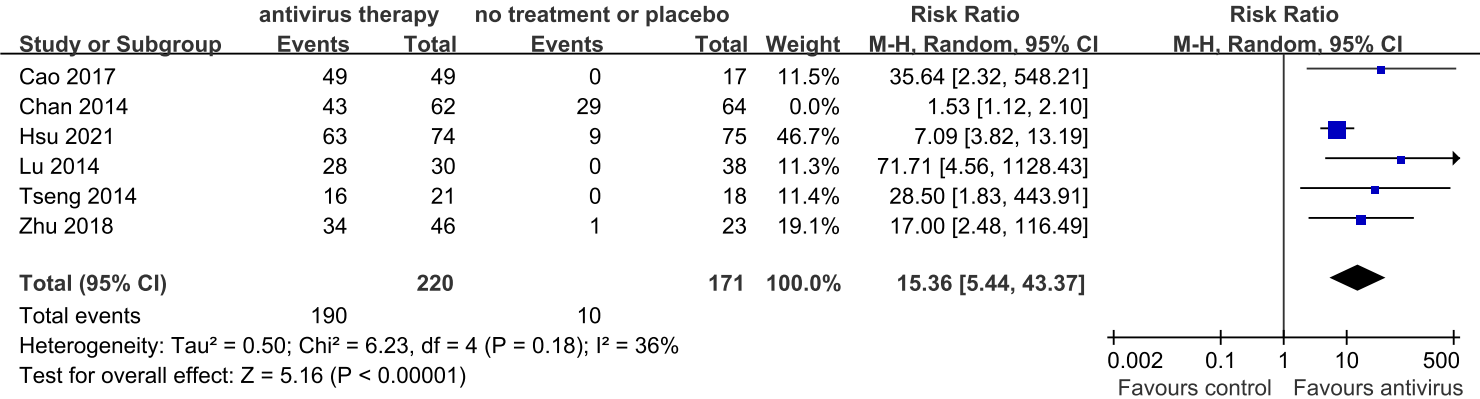

Supplement: Supplementary Materials — Supplementary Figure 1: risk difference was used for HBsAg loss under subgroup analysis stratified by therapeutic regimen with or without IFN. CI, confidence intervals. The size of square represents the weight of each study, and the vertical dotted line represents the pooled rate. Supplementary Figure 2: risk difference was used for HBsAg seroconversion under subgroup analysis stratified by therapeutic regimen with or without IFN. CI, confidence intervals. The size of square represents the weight of each study, and the vertical dotted line represents the pooled rate. Supplementary Figure 3: sensitivity analysis. (A) HBeAg loss. (B) HBV DNA undetectable. CI, confidence intervals. The size of square represents the weight of each study, and the vertical dotted line represents the pooled rate. [file 7499492.f1.zip › Supplementary Figure 3B.pdf]
